# Rapid resolution of generalized pustular psoriasis with upadacitinib

**DOI:** 10.1016/j.jdcr.2026.07.006

**Published:** 2026-07-09

**Authors:** Sonam Mistry, Mark Marchitto, Bruce Strober

**Affiliations:** a Central Connecticut Dermatology Research, Cromwell, Connecticut; b Rowan-Virtua School of Osteopathic Medicine, Stratford, New Jersey; c Department of Dermatology, Yale University, New Haven, Connecticut

**Keywords:** dupilumab, generalized pustular psoriasis, JAK inhibitor, janus kinase inhibitor, upadacitinib

## Introduction

Generalized pustular psoriasis (GPP) is a rare systemic cutaneous inflammatory disease characterized by recurrent episodes of widespread erythema, desquamation, and pustule formation.^1^ GPP is a severe and potentially life-threatening condition that carries mortality risks between 4% and 24%, making prompt diagnosis paramount to treatment success.^1^ Upadacitinib is a selective oral Janus kinase 1 (JAK1) antagonist that inhibits cytokine signaling in inflammatory disease pathways. Its role in the treatment of GPP has yet to be elucidated. Herein, we present a case of rapid GPP resolution with oral upadacitinib therapy.

## Case Report

**Fig 1:**
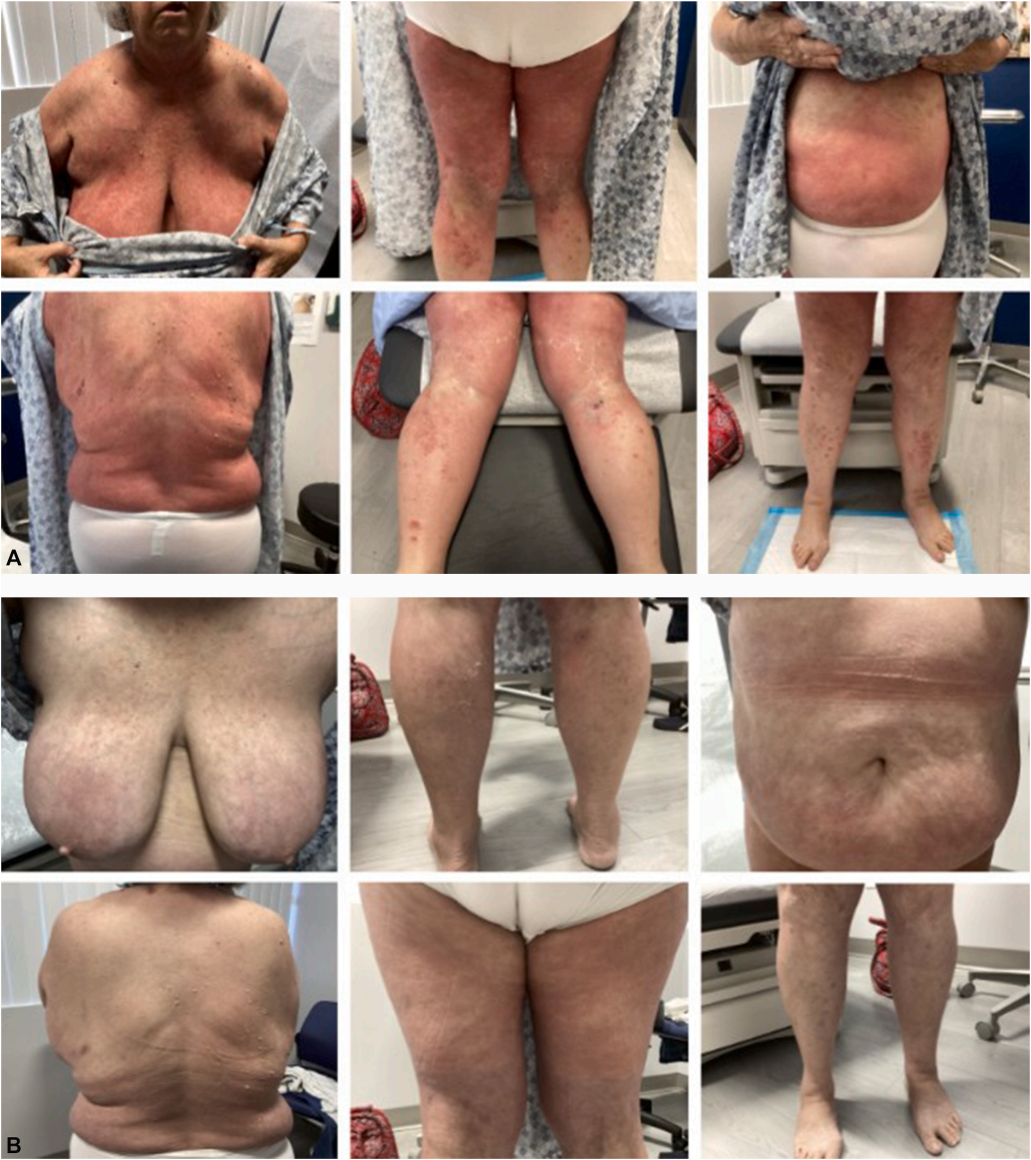
**A**, Initial presentation of the patient’s generalized pustular psoriasis prior to therapy. **B**, Presentation at the 2-wk follow up visit on upadacitinib therapy.

A 70-year-old female with a past medical history of childhood atopic dermatitis and asthma, as well as a strong family history of psoriasis, presented to our clinic for a subacute-onset rash that had progressed over the course of several weeks. Prior to presenting to our clinic, the patient was seen by an outside dermatology provider and prescribed topical corticosteroids, topical calcineurin inhibitors, oral antibiotics, oral terbinafine and several courses of oral steroids. Eventually the patient was initiated on dupilumab for suspected atopic dermatitis; after approximately 3 months of dupilumab therapy, her eruption worsened markedly. The patient was then referred by her primary care physician to our dermatology clinic for a second opinion. Upon initial examination, the patient presented with erythroderma, diffuse pustules, and desquamation affecting the scalp, face, trunk and extremities. She endorsed extreme pruritus and a painful burning sensation of the skin (Fig 1, *A*).

**Fig 2:**
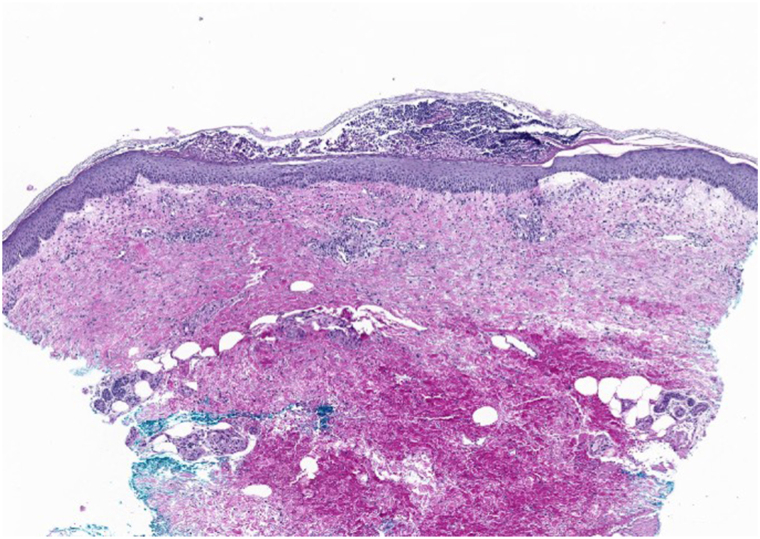
Histology demonstrating psoriasiform hyperplasia, thinned suprapapillary plates, diminution of the granular layer, neutrophilic infiltration within the epidermis and subcorneal pustule formation.

A diagnosis of GPP was clinically favored and subsequently confirmed by skin biopsy. Histopathology revealed psoriasiform hyperplasia, thinned suprapapillary plates, diminution of the granular layer, areas of confluent parakeratosis, dilated blood vessels in the papillary dermis, and neutrophilic subcorneal pustules (Fig 2). Laboratory evaluation was remarkable for an elevated white blood cell count of 14,200/uL consisting of predominantly neutrophils (9940/uL).

Dupilumab had been discontinued at the time of referral and remained discontinued for approximately 4 weeks prior to presentation, during which the eruption continued to progress. At her initial visit, treatment options were discussed. Although spesolimab is the only therapy specifically approved for GPP flares, it was not immediately available due to access and infusion-scheduling logistics; given the acute severity of the patient’s presentation and the need for prompt disease control, the patient was empirically initiated on oral upadacitinib 30 mg daily as a rapidly available agent for acute stabilization. Her condition rapidly resolved over a 2-week period. At the 2-week follow up visit, the patient reported a significant improvement in pain as well as pruritus (Fig 1, *B*). During follow-up, the patient disclosed a previously known history of heterozygous Factor V Leiden carriership, established through prior genetic testing performed independently of her dermatologic care. Given the thromboembolic risk associated with JAK inhibitors, upadacitinib was discontinued despite her favorable response, and spesolimab was initiated. While initial improvement was noted with spesolimab infusions and subsequent subcutaneous injections, the patient began to flare between maintenance doses. The patient was ultimately transitioned to secukinumab and her condition remains well-controlled.

## Discussion

GPP is an uncommon dermatitis with prevalence estimates ranging from 1.76 to 124 cases per million persons worldwide.^2^^,^^3^ The pathogenesis of GPP is multifactorial, involving both genetic susceptibility and environmental triggers, including viral infections, medications, and withdrawal of systemic corticosteroids.^4^ GPP is increasingly being recognized as a distinct clinical entity from plaque psoriasis, driven primarily by mutations in the interleukin (IL)-36 signaling pathway.^4^ Spesolimab, an IL-36 receptor antagonist, is the first and currently only Food and Drug Administration-approved medication specifically for GPP flares and prevention.^5^ In our experience, although the majority of patients with GPP respond favorably to IL-36 receptor antagonist therapy, a subset demonstrates a more robust clinical response to tumor necrosis factor, IL-17, or IL-23 inhibition.

Since the advent of biologic therapies, accumulating longitudinal evidence has demonstrated that inhibition of specific cytokine pathways may result in unanticipated clinical outcomes. For example, inhibitors targeting type II inflammatory pathways have been associated with the development of psoriasiform drug eruptions. This paradoxical phenomenon is thought to result from suppression of type II–mediated signaling, which may promote a relative shift toward type I and type III inflammatory pathways.

Dupilumab has been associated with the onset of GPP in several patients with a history of atopy.^6^, ^7^, ^8^ Notably, our patient had a documented history of childhood atopic dermatitis and asthma, and her GPP eruption developed after approximately 3 months of dupilumab therapy. Although a causal relationship cannot be definitively established, the temporal association suggests that dupilumab may have contributed to disease onset. Because dupilumab had been discontinued for approximately 4 weeks before upadacitinib was started, withdrawal of a putative trigger must be considered as a potential confounder of the observed improvement. However, several features argue against trigger withdrawal alone as the explanation: the eruption continued to worsen throughout the 4-week dupilumab-free interval, and clinical clearance occurred only after upadacitinib was introduced, with near-complete resolution within 2 weeks. Given dupilumab’s prolonged pharmacologic half-life, 4 weeks of discontinuation would already have produced substantial drug washout without clinical benefit, making the rapid response to upadacitinib more consistent with a direct therapeutic effect.

JAK inhibitors are increasingly utilized across dermatologic, rheumatologic, and gastrointestinal inflammatory disorders. No JAK1-selective inhibitor is currently Food and Drug Administration-approved for psoriasis, although deucravacitinib, an allosteric TYK2 inhibitor within the broader JAK family, is approved for moderate-to-severe plaque psoriasis. Nonetheless, JAK inhibitors have demonstrated potential efficacy in the management of acute GPP flares.^9^, ^10^, ^11^ Prior reports have described improvement of GPP with upadacitinib.^9^^,^^10^ Our case is distinguished by several features: dupilumab as a probable trigger, the exceptional speed of response to upadacitinib, and a real-world constraint—incidentally recognized Factor V Leiden carriership—that limited continuation of an otherwise effective JAK inhibitor.

JAK inhibitors may be effective in GPP through their ability to broadly modulate cytokine signaling pathways central to disease pathogenesis. GPP is driven in part by dysregulation of the IL-36 axis, which amplifies downstream inflammatory cascades involving neutrophil recruitment and activation. These processes are further propagated by cytokines such as IL-6, IL-23, and interferons, many of which signal through the JAK-STAT pathway. Inhibition of JAK activity may therefore attenuate multiple proinflammatory signals simultaneously, reducing keratinocyte activation and neutrophilic inflammation.

In particular, selective JAK1 inhibition may suppress signaling from key cytokines implicated in pustular psoriasis while preserving some degree of immune function, potentially offering a favorable therapeutic balance. This broad mechanism of action may be especially advantageous in acute GPP flares, where rapid control of systemic inflammation is required. Additionally, JAK inhibitors may modulate immune dysregulation associated with other targeted therapies, further supporting their potential role in cases of paradoxical or treatment-refractory disease. We propose upadacitinib not as long-term maintenance therapy but as a rapidly available oral option for acute stabilization that can bridge to definitive treatment, as illustrated by this patient’s subsequent transition to spesolimab and ultimately secukinumab. However, further studies are needed to better define their efficacy, safety, and optimal positioning in the GPP treatment algorithm.

## Conflicts of interest

Dr Strober has served as a consultant (received honoraria) for AbbVie, Alumis, Almirall, Amgen, Apogee, Arcutis, Bristol-Myers-Squibb, CorEvitas, Dimagi, Immunovant, Johnson & Johnson, Leo, Eli Lilly, Neurocrine, Novartis, Oruka, Meiji Seika Pharma, Protagonist, Pfizer, UCB Pharma, Regeneron, Sanofi-Genzyme, selectION, Takeda. He possesses stock options in Mindera Health, selectION. He has served as a speaker for AbbVie, Arcutis, Eli Lilly, Incyte, Johnson & Johnson, Regeneron, and Sanofi-Genzyme and as a co-scientific director (consulting fee) for CorEvitas Psoriasis Registry. He serves as an investigator for CorEvitas Psoriasis and GPP Registries, Oruka, and Incyte. Author Mistry and Dr Marchitto have no conflicts of interest to declare.
